# Giant paratesticular liposarcoma: A rare case report and literature review

**DOI:** 10.1016/j.ijscr.2024.109386

**Published:** 2024-02-23

**Authors:** Indrianto Wiryo Pranoto, Tarmono Djojodimedjo, Dimas Panca Andhika

**Affiliations:** a Department of Urology, Faculty of Medicine Airlangga University, Surabaya, Indonesia; b Department of Urology, Dr. Soetomo General Academic Teaching Hospital, Surabaya, Indonesia; c Department of Urology, Universitas Airlangga Hospital, Surabaya, Indonesia

**Keywords:** Paratesticular tumor, Liposarcoma, Testiscular neoplasm

## Abstract

**Introduction:**

Paratesticular liposarcoma is a rare variant of genitourinary malignancy. This malignancy accounts for less than 12 % of all liposarcomas. Approximately 200 cases of paratesticular liposarcoma have been reported. Giant paratesticular liposarcoma sizing over 10 cm is rarer, with only a few reported cases. Due to the rarity of this disease, there are no standardized guidelines regarding its incidence, diagnostic, recurrence, and treatment.

**Case presentation:**

A 73-year-old male came to the hospital with a painless left scrotal mass three years ago. The patient had an ultrasound examination of the left scrotal, which proved a solid mass and hypervascular on the left testicular. Abdominopelvic computed tomography (CT) showed a solid-cyst masses, size ±16,6 × 9,6 × 18,2 cm, lobulated, contrast enhancement with no sign of metastatic disease. The patient had radical orchiectomy without any complications. Histopathological and immunohistochemistry examination (Vimentin, MDM2, dan CDK4) showed well-differentiated liposarcoma.

**Clinical discussion:**

Radical orchiectomy is the best curative therapy. Adjuvant chemotherapy and radiotherapy benefit is still inconclusive. The patient had followed up for two years after surgery found no recurrent mass and metastatic. The well-differentiated type has a better prognosis but has a high incidence of local recurrence if incompletely excised. The result showed that this approach produces excellent outcomes without any relapse.

**Conclusion:**

Giant Paratesticular Liposarcoma is a rare condition that can be managed by radical. Long-term follow-up is importance to observe the relapse of this malignancy.

## Highlights

• Paratesticular liposarcoma is a rare variant of genitourinary malignancy.
• A complete excision might a crucial step to prevent the recurrence.
• Adjuvant radiotherapy is required for unclear-margin resection.

## 1 Introduction

An uncommon kind of malignant tumor called paratesticular liposarcoma often develops in the tissue surrounding the scrotum, including the tunica vaginalis, epididymis, and other supportive tissues. The prevalence of paratesticular liposarcoma is less than 12 % of liposarcomas [1]. Approximately 7 % of scrotal tumors are paratesticular liposarcomas. 90 % of lesions in the paratesticular liposarcoma in the reproductive system originate from the spermatic cord [2]. Liposarcoma is one of the most prevalent type of sarcoma, one-third of cases are malignant [3]. Paratesticular sarcoma is rare, with only 200 cases reported in the medical [4].

The risk factors of paratesticular liposarcoma are still remain unknown, some evidence suggests that aging may be a major risk factor. In addition, left paratesticular liposarcoma seems to develop more often than right paratesticular [4]. The tumor's size may also vary; in rare situations, tumor larger than 10 cm are referred as “giant tumor”. There are no established standards for its incidence, diagnostic, recurrence, or treatment due to the disease's rarity. Therefore, the majority of the diagnostic and treatment suggestions are based on prior case reports [5]. We discussed a giant paratesticular liposarcoma in this case report followed the SCARE Guideline checklist [6].

## 2 Case presentation

A 73-year-old male presented symptoms of a left testicle that had been becoming bigger for a year. The patient reported having a quail egg-sized lump in the left testicle for the last three years. There was no prior history of scrotal injuries or testicular undescensus. The patient had lipoma excision surgery on the right shoulder twelve years before admission to hospital.

**Fig. 1:**
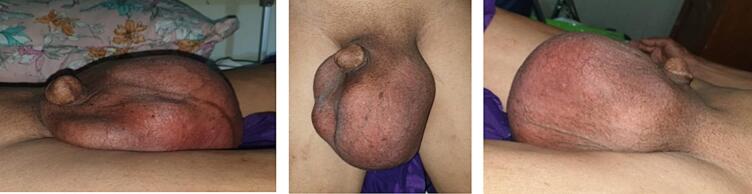
Clinical presentation.

Physical examination of the scrotum demonstrated a normal examination on right testicular, but in left scrotal found a mass about 15 × 10 cm, solid, firm, and moveable, as shown in Fig. 1. Further examination showed that hemoglobin was 15.4 g/dL. Moreover, the testicular tumor marker test were normal Lactate Dehydrogenase (LDH) (173 U/L), normal Human Chorionic Gonadotropin (HCG) (0.3 mIU/mL), and normal Alpha-Feto Protein (AFP) (4.9 ng/mL).

**Fig. 2:**
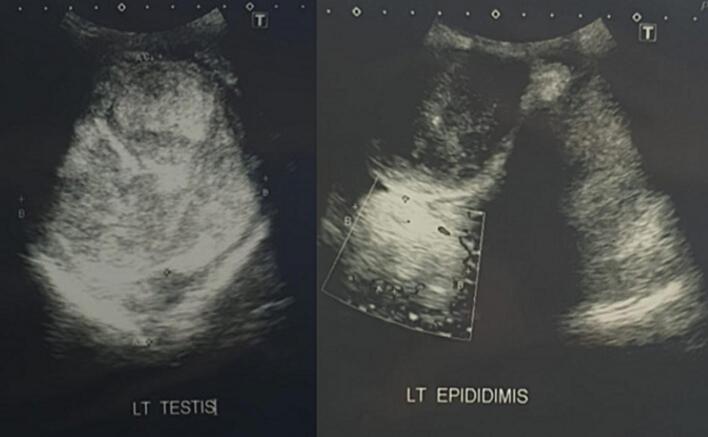
Testicular ultrasonography with doppler.

**Fig. 3:**
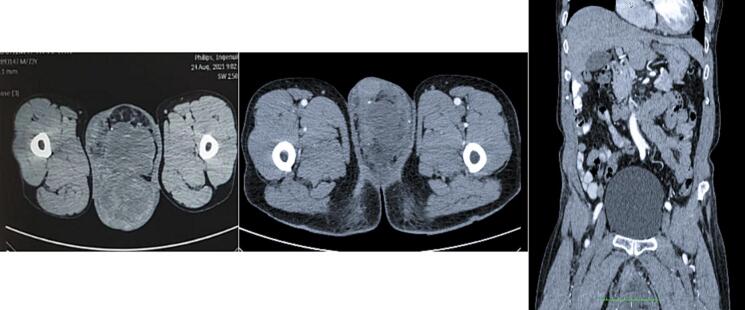
Abdominal and Pelvic CT-Scan showed an enhanced contrast effect in the lesions.

The testicular ultrasonography with Doppler showed a hypervascular mass that looked to nearly fill the left. This mass, about 10.5 × 9.9 × 8 cm, was suspected of infiltrating the left epididymis, as shown in Fig. 2. Despite this result, the right testicle was normal. After the ultrasonography, we performed abdominal and pelvic CT-scan with contrast, which showed an enhanced contrast in solid (32 HU) and cystic (3–25 HU) mass, lobulated, about 16.6 × 9.6 × 18.2 cm. There was subcentimeter lymph enlargement in the right inguinal, left inguinal, mesenteric, right para iliac, and left para iliac, as shown in Fig. 3.

**Fig. 4:**
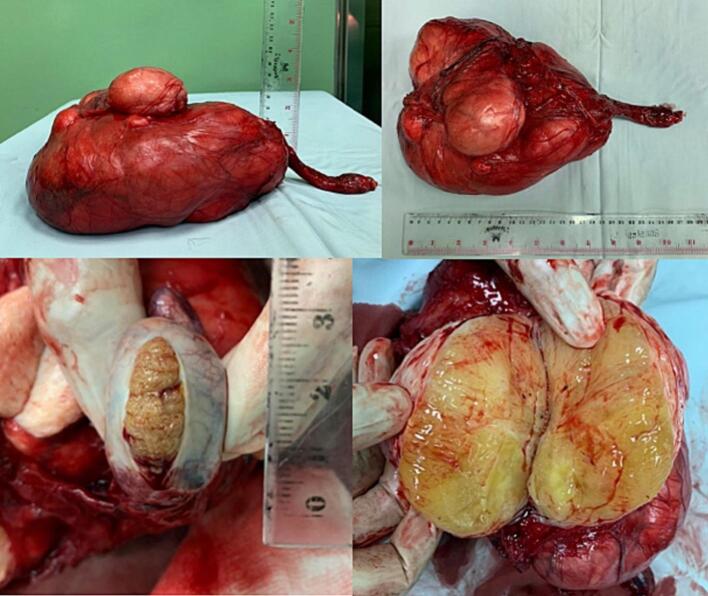
The resection of the testis showed a liposarcoma.

**Fig. 5:**
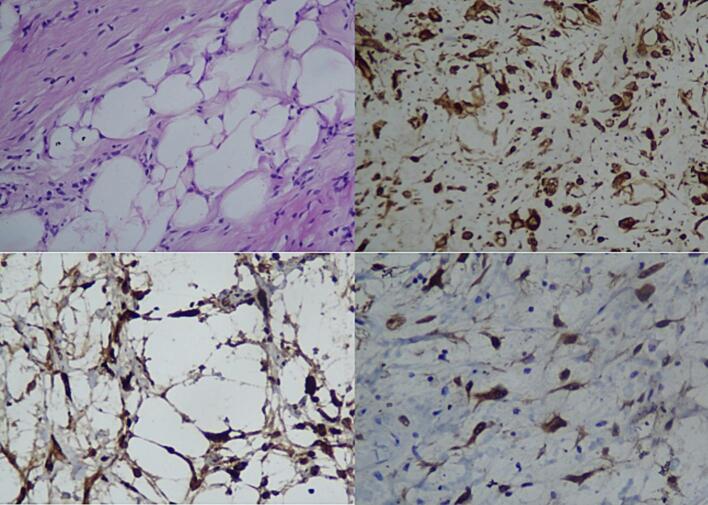
Histopathology and Immunohistochemical result. (a.) well-differentiated liposarcoma 200× High Power Field (HPF); (b.) Vimentin Positive 400× HPF; (c.) MDM2 Positive 400× HPF; (d.) CDK4 Positive 400× HPF.

We performed a left radical orchiectomy by inguinal approach with high ligation of the spermatic cord, which is disected proximal to the internal inguinal ring. The surgical was done by ligating and dividing the vas deferens from the cord. There were no cystic lesions and adhesions to the tissue around the tumor, about 19x9x12 cm, allowing for a clean excision, as presented in Fig. 4. The histopathology examination showed well-differentiated liposarcoma without infiltration. The liposarcoma showed a mixture of normal-appearing testicular tissues intermixed with atypical adipocytes. The atypical cells are hyperchromatic, pleomorphic and irregular. Therefore, we performed an immunohistochemistry analysis to confirm the histopathology, consisting of Vimentin, MDM2, and CDK4. The result was a well-differentiated liposarcoma, in which no infiltration occurs, as shown in Fig. 5.

## 3 Clinical discussion

Paratesticular tumors are scrotal tumors that did not originate from testicles. The tunica vaginalis, spermatic cord, epididymis tissue, and other supporting tissues may cause this tumor. Paratesticular liposarcoma is an extremely rare, and only around 200 case reports have been reported. This cancer represents 12 % of all sarcomas, and 90 % of paratesticular lumps are sarcomas, with less than one-third of them becoming malignant [5,7]. Lipomas, adenomatoid tumors, and leiomyomas are among the benign paratesticular masses. Liposarcoma, rhabdosarcoma, and leiomyosarcoma are the three most common types of malignant sarcomas, however, differentiated sarcoma or malignant fibrous histiocytoma may also occur. The majority of paratesticular sarcomas originate from the spermatic cord [8].

Some studies have shown that age is one of the risk factors for this tumor. The median age for this tumor was 62 years old, ranging from 15 to 89 years old. About 51.7 % of this malignancy occurs in the left paratesticular, with a median size of about 9.5 cm [9]. Another study said that this tumor most often afflicts adult patients between the ages of 50 to 60 years old, while it may affect person as young as 16 [10].

The pathogenesis of lipoma becoming liposarcoma remains unknown. One of the complaints associated with this cancer is tumors greater than 10 cm, which are usually referred to as enormous or giant tumors. According to Mouden et al., a case of a large paratesticular liposarcoma measuring 15x7x17 cm was documented [1]. Extensive excision is the current choice of treatment for paratesticular liposarcoma, according to many studies [11]. In this case, the patient had a left radical orchiectomy. A hypervascular solid mass almost filled the whole testicle was found at the time of the procedure. A clean excision was possible, there were no adhesions to the surround tissue.

Two types of histopathological examination that are often used are histopathology and immunohistochemistry. Histopathological examination can analyze the characteristics and type of liposarcoma [12]. In this case, histological and immunohistochemical examination revealed a well-differentiated liposarcoma. According to study by Köseolu and Yörükolu, the information obtained by histopathological examination may help in the development of appropriate treatment and management as well as in creating a more accurate diagnosis [13].

Diagnosis is mandatory for every lump or mass arising in the body at any location, particularly those rapidly growing and exceeding 5 cm in dimension, before initiating any active treatment, including surgical resection. Moreover, staging should consistently include a chest CT scan. Fundamentally, surgery for soft tissue sarcomas should be performed with compartmental resection whenever possible. In the case of spermatic cord liposarcoma, this should encompass not only the testicle, spermatic cord structures, and the mass but also extend to the inguinal canal [14].

Moreover, preventive action is the routine and thorough supervision of postoperative patients, which involves follow-up that includes physical examinations, blood tests, and appropriate imaging scans [12]. The follow-up becomes essential for the long-term monitoring of patients to monitor the cancer progressivity and recurrency [15]. Routine physical examinations, which would involve inspecting the testicles, scrotum, and surrounding areas, would be a good follow-up in this case. Imaging studies, such as CT-scan or ultrasonography, may also be used to evaluate the risk of recurrence or metastasis to other organs [16,17]. We performed the routine testicular ultrasonography examinations for this case at 6, 12, and 24 months after surgery and found no recurrence masses or metastases.

Adjuvant radiotherapy is the standard approach for post-surgical resection of high-grade, large or marginally excised on low-grade tumor. However, it was not required for clear-margin resection. The effectiveness and safety of adjuvant chemotherapy remain unclear but can be considered for individual patients with potentially chemosensitive subtypes [18].

The recurrence-free survival rate of those who had high inguinal resection was noticeably greater when compared to individuals who had tumors removed,. Additionally, those who had positive margins under the microscope showed a noticeably higher risk of recurrence, 55 % of cases, than those who had negative margins. In the subgroup analysis of those who had positive margins, the potential impact of postoperative adjuvant radiotherapy on the recurrence-free survival rate was not discovered to be statistically significant [19].

## 4 Conclusion

Giant paratesticular liposarcoma is a rare case, which can be treated with radical orchiectomy. Performing a complete excision that removes all tumor tissue to prevent the recurrence of liposarcoma. Follow-up that includes physical examinations, blood tests, and appropriate imaging scans are also essential.

## Consent

The informed consent was written by the patient in the Indonesian language for further publication of this case report anonymously. A copy of the written consent is available for review by the Editor-in-Chief of this journal on request.

## Ethical approval

Ethical approval has been acquired in this study by Health Research Ethics Committee of Dr. Soetomo General-Academic Hospital, Surabaya, Indonesia.

## Funding

This research did not receive any specific grant from funding agencies in the public, commercial, or not-for-profit sectors.

## Author contribution

Indrianto Wiryo Pranoto: Conceptualization; Formal analysis; Funding acquisition; Investigation; Methodology; Project administration; Resources; Supervision; Validation; Visualization.

Tarmono: Roles/Writing - original draft; Writing - review & editing, Conceptualization; Formal analysis; Investigation; Methodology; Project administration; Visualization.

Dimas Panca Andhika: Performed the procedure, Data curation; Funding acquisition; Investigation; Resources; Supervision; Validation; Visualization.

## Guarantor

Indrianto Wiryo Pranoto

## Research registration number

1. Name of the registry: N/A
2. Unique identifying number or registration ID: N/A
3. Hyperlink to your specific registration (must be publicly accessible and will be checked): -

## Conflict of interest statement

None.
